# T cell exhaustion is associated with cognitive status and amyloid accumulation in Alzheimer’s disease

**DOI:** 10.1038/s41598-023-42708-8

**Published:** 2023-09-22

**Authors:** Jason M. Grayson, Samantha M. Short, C. Jiah Lee, Nuri Park, Caitlyn Marsac, Alessandro Sette, Cecillia S. Lindestam Arlehamn, Xiaoyan I. Leng, Samuel N. Lockhart, Suzanne Craft

**Affiliations:** 1 Department of Microbiology and Immunology, Wake Forest University School of Medicine, 525 Wake Forest Biotech Place, 525 Patterson Avenue Room 2N051, Winston-Salem, NC 27101 USA; 2 Department of Internal Medicine-Geriatrics, One Medical Center Boulevard, Wake Forest University School of Medicine, Winston-Salem, NC 27157 USA; 3 La Jolla Institute for Immunology, 9420 Athena Circle, La Jolla, CA 92037 USA; 4 Department of Biostatistics and Data Science, One Medical Center Boulevard, Wake Forest University School of Medicine, Winston-Salem, NC 27157 USA

**Keywords:** CD4-positive T cells, CD8-positive T cells

## Abstract

Studies over the last 100 years have suggested a link between inflammation, infectious disease, and Alzheimer’s Disease (AD). Understanding how the immune system changes during the development of AD may facilitate new treatments. Here, we studied an aging cohort who had been assessed for AD pathology with amyloid positron emission tomography and cognitive testing, and conducted high dimensional flow cytometry on peripheral blood mononuclear and cerebrospinal fluid cells. Participants were assigned a classification of being amyloid negative cognitively normal, amyloid positive cognitively normal (APCN), or amyloid positive mild cognitive impairment (APMCI), an early stage of AD. We observed major alterations in the peripheral innate immune system including increased myeloid and plasmacytoid dendritic cells in the blood of APMCI participants. When the adaptive immune system was examined, amyloid positive participants, regardless of cognitive status, had increased CD3^+^ T cells. Further analyses of CD4^+^ and CD8^+^ T cells revealed that APMCI participants had an increase in more differentiated phenotype T cells, such as effector memory and effector memory CD45RA expressing (TEMRA), compared to those with normal cognition. When T cell function was measured, we observed that T cells from APCN participants had increased IFNγ^+^GzB^-^ producing cells compared to the other participants. In contrast, we demonstrate that APMCI participants had a major increase in T cells that lacked cytokine production following restimulation and expressed increased levels of PD-1 and Tox, suggesting these are exhausted cells. Rejuvenation of these cells may provide a potential treatment for AD.

## Introduction

AD is a neurodegenerative disease afflicting 6 million Americans^1^ and their care costs the US economy over $300 billion per year^2,3^. It is characterized by memory and other cognitive declines and the presence of Aβ plaques, neurofibrillary tangles (NFT), and increased inflammation in the central nervous system (CNS)^4^. There is no cure for this disease^5^. Thus, new treatments and approaches are needed as the number of adults over age 65 dramatically increases in the US.

Many studies over the last 100 years have established a link between inflammation, infectious disease, and AD. The first links to inflammation and AD were established when post-mortem brain analyses revealed sustained inflammatory infiltrates^6,7^ with amyloid plaques and NFT. Further illustration of the role of inflammation in cognitive decline is provided by nonsteroidal anti-inflammatory drugs (NSAID) usage: adults who periodically use NSAIDs have a lower risk of dementia or AD^8^, although use of NSAIDs in clinical trials has had mixed results^9^. The link between cognition and infection has also long been recognized. Acute infections including sepsis, meningitis and COVID can induce transient cognitive changes^10^. The relationship between chronic infection and long-term cognition is more recently recognized^11,12^. Most humans are exposed to 6–8 human herpesviruses through early adulthood^13–17^, and these viruses generate strong T cell responses that prevent reactivation. As we age, virus reactivates and replicates, causing systemic inflammation which could affect cognition^18^. Support for this idea is multi-faceted. Studies in the 1990s documented herpes simplex virus-1 (HSV-1) DNA in the brains of deceased AD patients^19,20^. In animal models of AD, recurrent HSV-1 infection can drive neurodegeneration and cognitive defects^21^. In humans, studies of zoster ophthalmicus patients from Taiwan provide insight^22,23^. Patients that were given anti-herpetic drugs have a twofold decrease in their incidence of senile dementia. Recently, studies have found increased human herpesvirus 6 (HHV6) DNA in the brains of Alzheimer’s patients^24^. While herpesviruses may be the dominant contributor to infection-linked inflammation driving AD, other pathogens also play a role. Studies have linked AD development to presence of bacterial and parasitic pathogens such as *Porphyromonas gingivalis*^25^, *Chlamydia pneumoniae*^26^, and *Toxoplasma gondi*^27^ in the brain. The common theme of all these pathogens is that they are chronic infections and can be carried throughout life. Further support for the idea that infection-linked inflammation is critical for AD development is provided by the finding that periodic influenza^28–30^, zoster^31^ or BCG^32,33^ vaccinations are associated with a decreased risk of AD in multiple populations across the world. Whether this is due to specific protection against their cognate pathogens, or non-specific “immune training” is not clear. Furthermore, studies by Roy and colleagues^34^ demonstrated one of the key downstream cytokines produced during infection, type I interferon, is induced in animal models of AD. Taken together these observations raise the issue of why don’t all ageing individuals, who are infected with these chronic pathogens develop AD? Thus understanding how the immune system is perturbed during the development of AD is critical.

In the current study we determined how the peripheral immune system was altered in an aging cohort at presymptomatic and early symptomatic developmental stages of AD. Using high dimensional flow cytometry on PBMC and CSF cells combined with amyloid PET imaging and cognitive testing, we identified multiple changes in the innate and adaptive immune systems that occur following the onset of amyloid pathology, and subsequent onset of cognitive symptoms. We found that amyloid positive mild cognitive impairment (APMCI) participants contained more differentiated phenotype CD4^+^ and CD8^+^ T cells than amyloid negative cognitively normal participants (ANCN). When function was interrogated, amyloid positive participants with normal cognition (APCN) had increased numbers of cytokine producing T cells, while those with mild cognitive impairment had a pronounced increase in exhausted T cells. These findings suggest that approaches to rejuvenate T cell function may be potential treatments for AD.

## Results

### Altered innate and adaptive immune cell populations in amyloid positive aging participants

**Figure 1:**
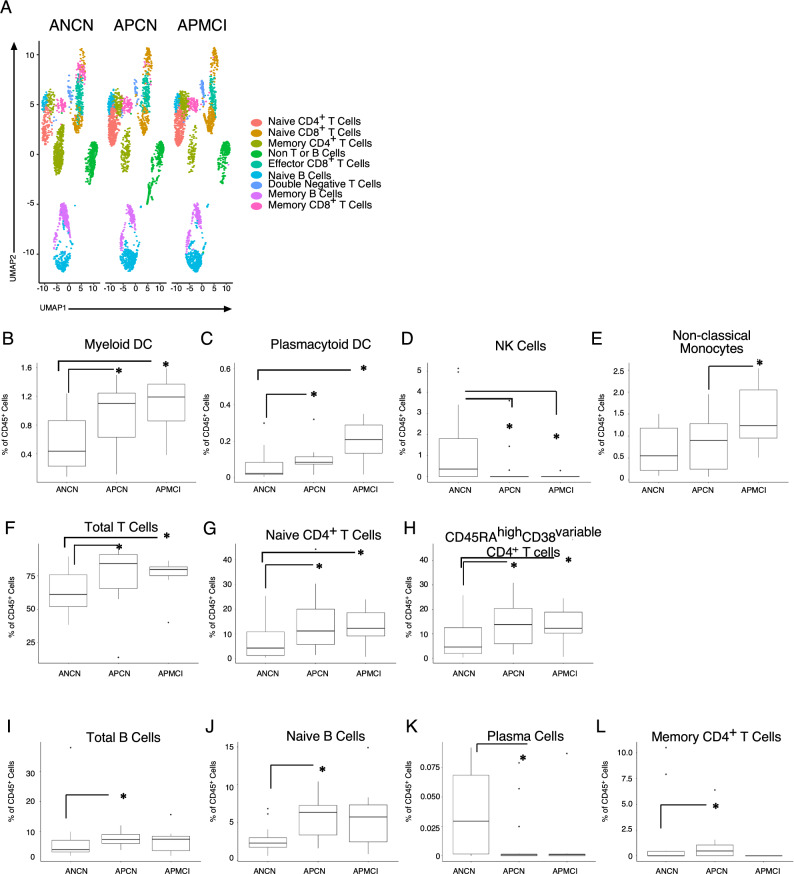
Altered innate and adaptive immune cell populations in amyloid positive aging participants. Peripheral blood mononuclear cells (PBMCs) from participants were stained with a pan-immune antibody cocktail and viable, CD45^+^ events were analyzed and (**A**) uniform manifold and approximation projection (UMAP) dimensionality reduction and Phenograph clustering was performed. Automated clusters were condensed and annotated by user expertise. In a separate analysis using prior immunological knowledge (**B**) Myeloid Dendritic cells, (**C**) Plasmacytoid Dendritic cells, (**D**) Natural killer cells, (**E**) Non-classical monocytes, (**F**) Total T Cells, (**G**) Naïve CD4^+^ T cells, (H)CD4^+^CD45RA^high^CD38^variable^ T cells, (**I**) Total B cells, (**J**) Naïve B Cells, (**K**) Plasma B cells were quantitated and the percentage of viable CD45^+^ cells is plotted with the median and interquartile ranges shown. Cerebrospinal fluid was analyzed (**L**) and the percentage of memory CD4^+^ T cells was plotted. The percentage of viable CD45^+^ cells is plotted with the median and interquartile ranges shown. Thirty-eight participants were analyzed (ANCN:16, APCN:15, ANMCI:7).* indicates *p*-value ≤ 0.05 in Wilcoxon testing.

To understand how the immune system impacts the development of Alzheimer’s Disease (AD), blood and CSF samples were obtained from 43 participants enrolled in the Wake Forest Alzheimer’s Disease Research Center, who had been intensively assessed by experts with cognitive testing and amyloid PET imaging. Samples were subjected to peripheral blood mononuclear (PBMC) and cerebrospinal fluid cell (CSFc) isolation. The demographic characteristics of our cohort are presented in Supplemental Fig. I. The mean age was 70.4 years with a BMI of 27. Importantly these characteristics were similar across all 3 groups. Based on mini mental status exam (Supplemental Fig. II, panel A) amyloid PET imaging (B) or CSF ELISA (C) or measurement of Aβ42/40 ratio (D), participants were assigned a classification of amyloid negative cognitively normal (ANCN), amyloid positive cognitively normal (APCN), or amyloid positive mild cognitive impairment (APMCI). Brain regions examined are listed in Supplemental Fig. III. PBMCs and CSFcs were stained with a pan-immune antibody panel that allows a wide range of range of cells across the innate and adaptive immune systems to be analyzed (Supplemental Figs. IV and V). Data from viable CD45^+^ cells were read into R and then dimensionality reduction was performed using the uniform manifold and approximation projection (UMAP) algorithm. Additionally, R phenograph was applied to the dataset and clusters were manually annotated to reflect cellular identity. Figure 1A shows the results of plotting UMAP values for each classification. In the top of the plots, T cells separate from both innate and B cells. Importantly, across all three components of the immune system there are differences according to amyloid and cognitive classification. Data were then analyzed manually using the gating strategy described in Supplemental Fig. V. In the blood we observed an increase in myeloid dendritic cells with amyloid positivity regardless of cognitive status (Fig. 1B). When plasmacytoid dendritic cells were quantitated (Fig. 1C) an increase was again observed in amyloid positive participants, but this was more pronounced in those with mild cognitive impairment. These changes contrast with natural killer cells (Fig. 1D) that were decreased below the detection limit in both groups of amyloid positive participants. Finally, we observed a modest increase in non-classical monocytes (Fig. 1E) in APMCI participants. Taken together these results demonstrate changes in the innate immune system early during the progression to AD.

We also examined how the adaptive immune system was impacted at early developmental stages of AD. Here we observed an increase in total CD3^+^ T cells in amyloid positive participants (Fig. 1F). When naïve (Fig. 1G) and effector-like CD38^variable^ CD4^+^ (Fig. 1H) T cells were measured both cognitively normal and cognitively impaired amyloid positive participants had increased numbers. Finally, we quantitated the changes in the B cell compartment and observed a modest increase in total B cells in APCN (Fig. 1I), that appeared to be in the naïve/mature B cell compartment (Fig. 1J). Interestingly, total blood plasma cells were below the detection limit in amyloid positive participants. When we measured cells present in the CSF, only memory CD4^+^ T cells were significantly increased in APCN (Fig. 1L). Thus the early progression to AD is accompanied by broad changes across the peripheral immune system.

**Figure 2:**
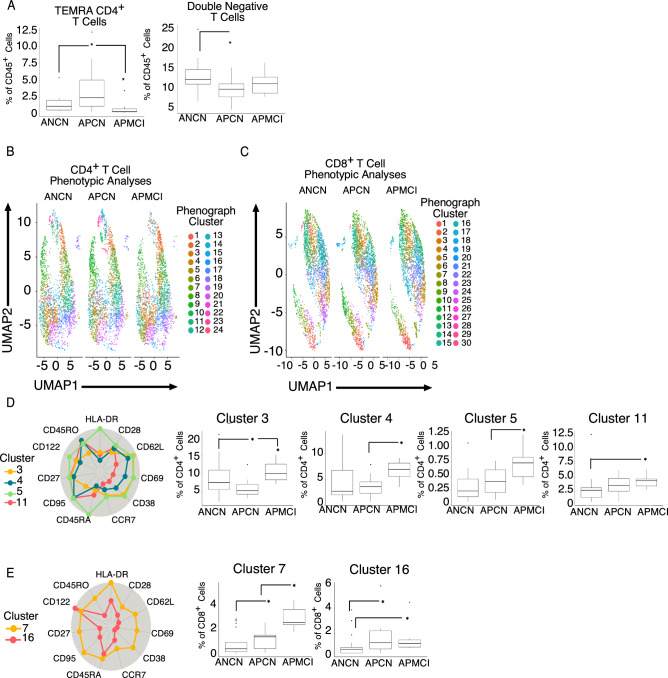
Increased T cell activation in amyloid positive aging participants. Peripheral blood monuclear cells (PBMCs) from participants were stained with a T cell differentiation antibody cocktail and viable, CD45^+^CD3^+^ events were analyzed and (**A**) CD4^+^ TEMRA and (**B**) CD4^-^CD8^-^ Double Negative T cells as determined by user expertise were plotted. (**C**) CD4^+^ or (**D**) CD8^+^ T cells were exported and uniform manifold and approximation projection (UMAP) dimensionality reduction and Phenograph clustering was performed. Automated clusters are indicated in the legend. Clusters with statistically significant differences in the (D) CD4^+^ or (E) CD8^+^ T cell compartment are plotted. The percentage of viable CD4^+^ or CD8^+^ T cells is plotted with the median and interquartile ranges shown. Forty participants were analyzed (ANCN:17, APCN:15, ANMCI:7). * indicates *p*-value ≤ 0.05 in Wilcoxon testing.

Based on our results and other studies^35^ there are suggestions that T cells are altered in AD development. To address this, we stained samples with an antibody panel that demarcates naïve, effector and memory T cells subsets (stem cell memory, central memory, effector memory, effector memory RA positive (TEMRA)). Figure 2A shows that manually-gated (See Supplemental Fig. VI) CD4^+^ TEMRA memory cells are increased in APCN participants but they decline in APMCI. Interestingly, amyloid positive participants regardless of cognitive status had a decrease in CD3^+^CD4^-^CD8^-^ double negative (DN) T cells. Because of the large number of variables, we performed UMAP dimensionality reduction on total CD4^+^ (Fig. 2B) and CD8^+^ (Fig. 2C) T cells. This was combined with Phenograph clustering to discern how phenotype changed with AD progression. In the CD4^+^ population we observed a dip in CD45RA^low^CD45RO^high^HLADR^int/high^CD28^high^CD62L^high^CD38^low^CCR7^low^CD27^high^CD122l^ow^CD95^low^ cells ((cluster 3) similar to central memory phenotype) in APCN compared to the other groups (Fig. 2D). When cells with a CD45RA^high^CD45RO^high^HLADR^high^CD28^int^CD62L^high^CD69^high^CD38^int^CCR7^low^CD27^high^CD122^high^CD95^high^ (cluster 5) were quantitated, we observed an increase in APMCI participants. Additionally, effector-memory like cells (clusters 4 and 11) were also increased with amyloid positivity, particularly in APMCI patients. When CD8^+^ T cells were examined, we also noted an increase in cells with characteristics of both TEMRA and effector memory T cells in APMCI. Interestingly these cells could be distinguished by expression of CD95 and HLA-DR (clusters 7 vs. 16, Fig. 2E). Thus, the peripheral T cell compartment of participants experiencing mild cognitive impairment is marked by an increase in differentiated phenotype T cells.

**Figure 3:**
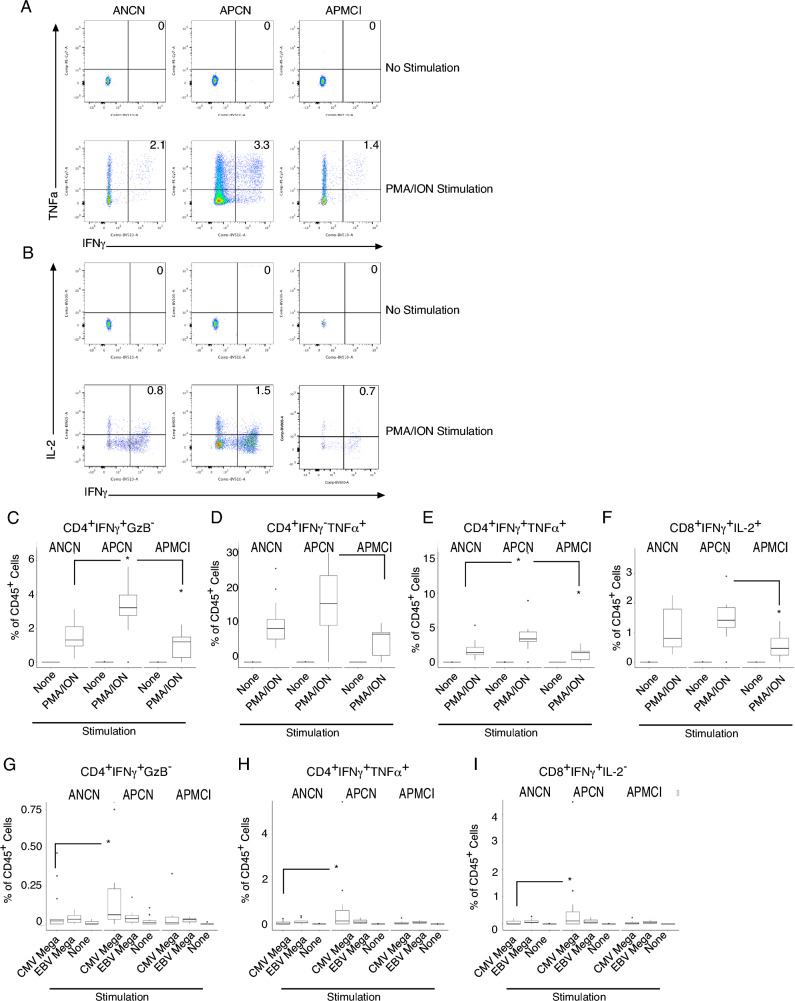
Increased T cell cytokine production in amyloid positive cognitively normal aging participants. Peripheral blood monuclear cells (PBMCs) from participants were stained with a T cell function antibody cocktail and viable, CD45^+^CD3^+^ events were analyzed and gated on (**A**) CD4^+^ or (**B**) CD8^+^ T cells and the indicated cytokines are plotted. The number in the upper right quadrant is the percent of viable CD45^+^ cells. CD4^+^ T cells were examined for cytokine production following PMA and ION stimulation and (**C**) IFNγ^+^GranzymeB^-^ (**D**) IFNγ^-^TNFα^+^ or (**E**) IFNγ^+^TNFα + as a percentage of viable CD45^+^ cells is plotted with the median and interquartile ranges shown. (**F**) CD8^+^ IFNγ^+^IL-2^+^ cells were quantitated after PMA and ION stimulation. (**G**) CD4^+^ or (**H**, **I**) CD8^+^ cells were stimulated with the indicated peptide mega-pool and (**G**) IFNγ^+^GranzymeB^-^ (**H**) IFNγ^-^TNFα^+^ (**I**) IFNγ^+^IL-2^-^ are plotted. Thirty-two participants were analyzed (ANCN:13, APCN:12, ANMCI:7). * indicates *p*-value ≤ 0.05 in Wilcoxon testing.

To address how this change in differentiation impacted function, we performed intracellular cytokine staining (Fig. 3A). PBMCs were stimulated for 5 h at 37 °C with phorbol myristate acetate and ionomycin (PMA/ION) to induce cytokine production. APCN participants had a significant increase in CD4^+^ IFNγ^+^TNFα^+^ T cells compared to the other two groups (Fig. 3A). Similar results were also observed with CD8^+^IFNγ^+^IL-2^+^ cells in this group (Fig. 3B). When results from multiple participants were quantitated, we observed an increase in IFNγ^+^GzB^-^ (Fig. 3C), IFNγ^-^TNFα^+^(Fig. 3D), and IFNγ^+^TNFα^+^(Fig. 3E) CD4^+^ T cells. We also observed an increase in CD8^+^IFNγ^+^IL-2^+^ (Fig. 3F) cells in APCN participants, but a decrease in APMCI subjects. Because PMA/ION is a very strong stimulus, we aimed to determine cytokine production from antigen-specific T cells. To address this, we stimulated cells for 5 h at 37 °C with peptide “mega”-pools composed of all possible combination of viral peptides to bypass concerns about HLA allele binding^36^. Here we compared cytokine production of Epstein-Barr virus (EBV) and Cytomegalovirus (CMV) specific CD4^+^ and CD8^+^-specific T cells. Similar to polyclonal cells we observed a statistically significant increase in CD4^+^ IFNγ^+^GzB^-^ cells in the CMV, but not EBV-specific cells (Fig. 3G). Here the trend was similar, but it was just above statistical significance. When CD8^+^ T cells were examined, we found that CMV-specific IFNγ^+^TNFα^+^ (Fig. 3H) and CD8^+^ IFNγ^+^IL-2^-^ (Fig. 3I) were increased in APCN participants. Thus, T cell cytokine production to PMA/ION or CMV-specific stimuli can distinguish cognitive status in amyloid positive participants which is a novel and important finding with implications for biomarker development.

**Figure 4:**
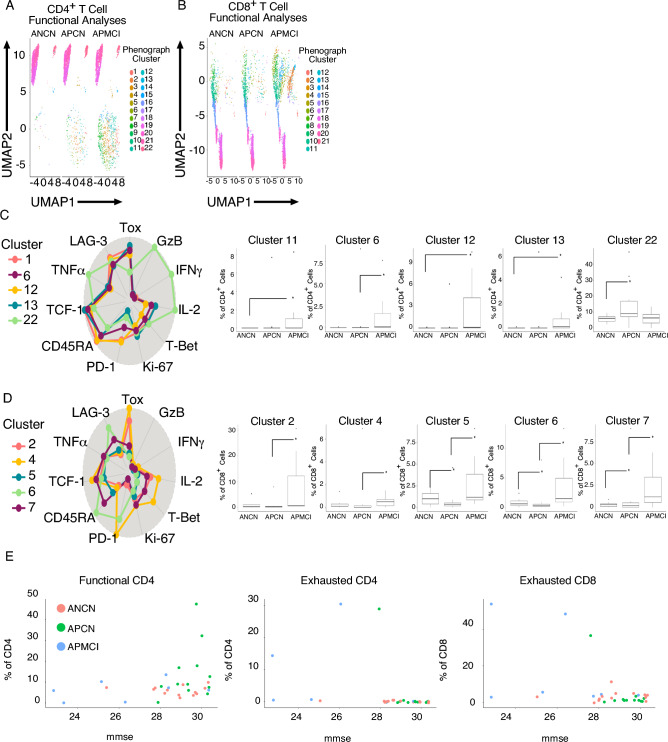
Increased T cell exhaustion in amyloid positive mild cognitive impairment aging participants. Peripheral blood mononuclear cells (PBMCs) from participants were stained with a T cell function antibody cocktail and viable, CD45^+^CD3^+^ events were analyzed and gated on (**A**) CD4^+^ or (**B**) CD8^+^ T cells and and uniform manifold and approximation projection (UMAP) dimensionality reduction and Phenograph clustering was performed. Automated clusters are indicated in the legend. Clusters with statistically significant differences in the (**C**) CD4^+^ or (**D**) CD8^+^ T cell compartment are plotted. The percentage of viable CD4^+^ or CD8^+^ cells is plotted with the median and interquartile ranges shown. (**E**) Exhausted and functional T cell clusters were condensed and plotted against MMSE score for each individual. Thirty-two participants were analyzed (ANCN:13, APCN:12, ANMCI:7). * indicates *p*-value ≤ 0.05 in Wilcoxon testing.

Because we observed a decrease in cytokine-producing T cells in APMCI participants we examined the expression of cytokines coupled with exhaustion markers and transcription factors. These data were then put into UMAP dimensionality reduction and Phenograph clustering. The results for CD4^+^ T cells are plotted in Fig. 4A. Similar results for CD8^+^ T cells are plotted in Fig. 4B. In both cases, there is a large population (Fig. 4A, bottom, Fig. 4B, top) in the amyloid positive participants that is substantially increased in those with mild cognitive impairment. When each subpopulation of CD4^+^ T cells (Fig. 4C) was analyzed, we observed an increase in APMCI participants of IFNγ^-^TNFα^-^IL-2^-^ Tox^+^TCF-1^+^T-Bet^low^cells that could be distinguished (clusters 1,6,12, and 13) by varying PD-1 expression. Similar to the manual analyses, clustering identified a population of IFNγ^+^TNFα^+^IL-2^+^ Tox^low^TCF-1^+/-^T-Bet^low^ cells functional cells that were increased in APCN participants (Cluster 22). CD8^+^ T cells displayed similar trends, i.e., there were five significant clusters identified that were increased in APMCI participants. All clusters lacked Granzyme B expression and had low or negative levels of IFNγ, TNFα and IL-2 (clusters 2, 4, 5, 6, and 7). Interestingly, the algorithm distinguished clusters based on their Tox, TCF-1 and PD-1 levels that were similar to classically exhausted T cells in cancer and HIV infection. Because our cohort was not balanced, we examined the effect of biological sex as a variable for our clustering analyses (Supplemental Fig. VII). When exhausted clusters of CD4^+^ (panel A) or CD8^+^ T cells were examined the same trends were observed in female only participants. Finally to understand the relationship between cognition and T cell exhaustion we combined the exhausted and functional CD4^+^ and CD8^+^ T cell clusters for each participant and then compared them with their mini mental status exam (MMSE) score. When functional CD4^+^ T cells were examined it is clear that APCN participants with the highest MMSE also had the greatest percentage of functional T cells (Figure E). When exhausted CD4^+^ or CD8^+^ T cells were compared it was also apparent that lowered MMSE was accompanied by an increase in exhausted T cells. Taken together these data argue the progression to MCI during early disease is accompanied by a dramatic increase in exhausted T cells.

## Discussion

In this study we examined immune system alterations early in the progression to Alzheimer’s Disease. We observed multiple changes across the peripheral innate and adaptive immune systems associated with amyloid and cognitive status within our aging cohort. In the innate immune system, we observed increased plasmacytoid and myeloid dendritic cells in amyloid positive participants, but these changes were particularly pronounced in those mild cognitive impairment. We also observed a decrease in total natural killer cells with amyloid positivity. When the adaptive immune system was examined, we observed increases in total T and B cells in amyloid participants. To further understand alterations in the T cell pool we used high dimensional flow cytometry to interrogate T cell differentiation and function. We observed an increase in differentiated CD4^+^ and CD8^+^ T cell phenotypes in amyloid positive participants with mild cognitive impairment. Surprisingly, we observed an increase in functional CD4^+^ and CD8^+^ T cells in amyloid positive cognitively normal participants, while those from amyloid positive mild cognitive impairment subjects had a dramatic increase in exhausted T cells. Importantly when T cell function was compared to cognitive status as determined by MMSE, patients with the lowest score had the highest number of exhausted cells.

Understanding how inflammation and the immune response control the development of AD is critical to develop new treatments. While AD is a disease of the brain, our results demonstrate changes of the immune system in the blood. The increases in both plasmacytoid and myeloid dendritic cells are suggestive of an ongoing response in amyloid positive participants regardless of cognitive status that precedes dementia. Here our results contrast with a prior study by Ciaramella and colleagues^37^ that showed no change in myeloid or plasmacytoid dendritic cells in MCI versus healthy participants. While the increase in dendritic cell subsets was suggestive of infection, our results with natural killer cells are intriguing. In both groups (AP) we detected very low levels of cells in circulation. One possibility is that NK cells may migrate from the blood to the brain to due to ongoing inflammation. In our analyses of CSF cells we did not observe a statistically significant difference in the number of NK cells, but they may reside in the brain parenchyma. While we could detect changes in the innate immune system reminiscent of infection, amyloid positivity was also accompanied by changes in adaptive immunity, particularly in CD4^+^ and CD8^+^ T cell phenotype and function. Interestingly, we observed an increase in naïve phenotype CD4^+^ T cells with amyloid positivity. One potential explanation could be that as part of an ongoing chronic response, new naïve T cells are generated, or other cell types migrate to peripheral tissues elevating the naïve T cell percentage in the blood.

Using both manual and algorithm guided analyses we observed major changes in T cell function and differentiation. Our manual analyses demonstrate that CD4^+^ TEMRA cells are increased in the circulation of APCN compared to either APCN or APMCI participants. These results confirm and extend the efforts of many laboratories to understand the role of T cells in AD. One of the first studies to perform immune profiling compared 45 healthy controls with AD patients and noted increased CD4^+^ but decreased CD8^+^ T cell percentage in the blood of AD patients^38^. Other investigators followed up on this finding and identified an increase in CD45RO-positive CD4^+^ T cells^39^. Speciale and colleagues observed a decrease in memory phenotype CD8^+^ T cells^40^. This contrasts with Larbi and colleagues who observed a decrease in CD4^+^ T and an increase in CD8^+^ T cells with AD^41^. While these initial studies provided a foundation, they had a relatively low number of antigens interrogated, and limited inclusion of patients early in the disease process, particularly those with mild cognitive impairment. Also these studies did not measure amyloid positivity. Recently Gate and colleagues^35^ studied a cohort of NC, MCI and AD participants and observed increased TEMRA CD8^+^ T cells in the blood, and cells from MCI and AD patients produced greater cytokines. They also observed expanded clones of Epstein-Barr virus-specific T cells in the cerebrospinal fluid of 2 AD patients. Other studies by Joshi and colleagues have demonstrated increased memory phenotype CD4^+^ T cells with reduced TCR diversity^42^. Using manually guided analyses, we observed an increase in CD4^+^, but not CD8^+^ TEMRA cells in amyloid positive participants. Why these differences were observed is not clear, but our study focused on patients early in the disease process, whereas Gate and colleagues included patients with advanced disease. Importantly we observed an increase that was associated with amyloid positivity and normal cognition arguing that the increase in differentiated T cell phenotypes start earlier than cognitive changes. When the high dimensional flow cytometry data for surface phenotype markers were used in unsupervised learning algorithms, multiple populations of differentiated phenotype (effector-like and effector memory-like) were increased with the transition to mild cognitive impairment. CD8^+^ T cell cluster 7 had increased HLA-DR expression, suggestive of recent stimulation, and was most pronounced in APMCI participants. However, differences in T cell function and transcription factor expression allowed us to clearly discern mild cognitive impairment from normal cognition in amyloid positive participants early in disease. We observed increased cytokine production in total CD4^+^ and CD8^+^ T cells from amyloid positive cognitively normal participants. Importantly we used multiple stimuli to activate T cells: PMA and ION, and antiviral peptides. Here we demonstrate the percentage of functional CMV-specific CD4^+^ and CD8^+^ T cells is increased in APCN participants. This contrasts with APMCI where the percentage had declined to levels below those in ANCN. By combining exhaustion markers and transcription factors an important trend was revealed. In amyloid positive participants a distinct population emerged. The cells (both CD4^+^ and CD8^+^) were very similar to exhausted cells first identified in LCMV-Clone 13 infection^43^, i.e., they expressed decreased cytokines upon restimulation and increased inhibitory receptors such as PD-1^44^. Wu and colleagues also noted increased PD-1 expression on PBMCs from AD patients^45^. They also had altered levels of TCF-1 and Tox-1 transcription factors which would decrease their proliferative and renewal potential^46–48^. These results are novel and may be critical in both understanding and treating AD.

Given the numerous links between infection, inflammation and AD our results suggest two models where T cells may be the nexus for disease. In the first, amyloid production is a response to simmering infections in periphery and brain with the multiple chronic pathogens all humans carry. Individuals who have strong T cell function control the replication of these pathogens and remain cognitively normal. This would explain why our APCN particpants, who have the most functional T cells still have the highest MMSE score. But in individuals who lose T cell function, chronic pathogens reactivate, overstimulate innate responses, particularly type I interferon production, potentially leading to cognitive impairment. This is the model we favor and suggests rejuvenation of T cells by immune checkpoint inhibitors and other treatments may be a plausible ex vivo therapy for AD. Indeed testing of immune checkpoint inhibitors in the 5X FAD mouse model of AD has shown promising results^49^. An alternative model posits that the cytokine production by the T cells while participants are cognitively normal drives the development of cognitive impairment. Support for this idea is provided by a recent study by Jorfi and colleagues that used in vitro cultured stem cell derived neurons, astrocytes, and microglia incubated with healthy PBMCs that showed increased microglial activation and inflammation driven by CD8^+^ T cells mediated by CXCR3 driven infiltration^50^. While these are important and interesting results there are two reasons that a protective rather than pathogenic role for T cells may be warranted. First, from a teleological point of view the increased function may be a form of resilience helping to stave off disease from chronic innate inflammation as we described earlier. The second, is that several studies have observed an increase in IFNγ that is associated with slower symptomatic progression in AD^51,52^. Discerning between these two models will require a longitudinal study to understand the exact temporal relationship between T cell function, exhaustion and cognitive function.

In conclusion, we demonstrate that the cognitive status of amyloid positive participants can be discerned by quantitating the number of exhausted CD4^+^ and CD8^+^ T cells. Amyloid positive participants with mild cognitive impairment also have increased differentiated T cells and myeloid and plasmacytoid dendritic cells in the blood. Taken together these observations provide a link between inflammation, infection, and cognitive decline. Importantly, they also suggest that many of the interventions being used to revive exhausted cells in cancer and chronic viral disease immunotherapy could be tested for efficacy in AD.

## Methods

### Study design

The study aimed to determine immune system alterations early during the development of Alzheimer’s Disease. The Wake Forest Alzheimer’s Disease Research Center ADRC includes ~ 600 adults for whom blood collection and cognitive/clinical evaluation occurs annually. Informed consent was provided by all subjects or their legal guardians. MRIs are conducted on all participants every two years, and CSF collection and ^11^C Pittsburgh Compound (PiB) amyloid and ^18^F AV1451 tau PET imaging are conducted in a subset of participants (n = 250) every 2 years. All participants receive extensive cognitive, clinical and medical assessment using a standardized protocol, the Uniform Dataset Version 3 (UDS3), developed and approved for all NIH ADRCs and are then diagnosed following expert interdisciplinary consensus conference. Participants are aged ≥ 55 years at entry and meet inclusion criteria for membership in 1 of 3 groups described below. Exclusion criteria include presence of a neurologic disorder other than AD, large vessel stroke, uncontrolled medical condition such as unstable cardiac disease, or cancer within 1 year.

The Normal Control group has no cognitive impairment, typically defined by scores on neuropsychological testing not worse than 1 standard deviation (SD) below the mean for age, education, and race, and Clinical Dementia Rating (CDR) of 0. The MCI group meets National Institute on Aging-Alzheimer’s Association (NIA-AA) criteria for MCI, adjudicated through a multidisciplinary consensus conference and defined by objective evidence of cognitive deficits on neuropsychological testing, and largely intact activities of daily living. The AD group consists of persons with mild dementia due to AD diagnosed with NIA-AA criteria, including a CDR = 0.5–1.0.

### Individuals and samples

Forty-three individuals were selected from the ADRC for this study. Thirty-eight individuals were selected for pan immune surface staining because both PBMC and CSFc were available. Thirty-two individuals were selected for intracellular cytokine staining. Blood samples and cerebrospinal fluid isolation were performed in the Wake Forest Clinical Research Unit. Blood samples were immediately processed by LymphoPrep gradient and then cryopreserved for future use. CSF cells were pelleted and cryopreserved for future use. Cells were thawed the night before, viability was assessed initially by trypan blue exclusion, and cells were stained with indicated antibodies.

#### MRI and PET imaging

MRI*.* Participants are scanned on a research-dedicated 3 T Siemens Skyra (32-channel head coil). High-resolution T1-weighted images are obtained with a fast gradient echo (GE) sequence: TR = 2300; TE = 2.98; 1 mm isotropic. T1 MRI scans were segmented using FreeSurfer v5.3 (https://surfer.nmr.mgh.harvard.edu) to generate target and reference regions used in PET processing (see below).

#### Aβ PET

Participants are injected with an i.v. bolus of ~ 10 mCi (370 MBq) (± 10%) [^11^C]PiB over 5-10 s, followed by 40-min uptake. CT is done prior to PET for attenuation correction. Emission images are acquired continuously for 40–70 min post-injection on a 64-slice GE Discovery MI DR PET/CT scanner. PET Images were reconstructed as multi-frame images, and motion correction was applied to the PET images, which were then averaged into a 3D image. ANTs (http://stnava.github.io/ANTs/) was used to estimate the transformation of participant CT scans to MRI space, and to apply the transformation to coregister PET images (in the same native space as CT) to MRI scans. Aβ + is classified using trained visual raters^53^ (Aβ burden is also quantified on PET using standardized uptake value ratio (SUVR; cerebellar GM reference^54,55^) averaged from a cortical meta-region of interest (ROI) sensitive to early AD^55,56^, using MRI-defined (FreeSurfer) brain regions (see Supplementary Table), and continuous Aβ + SUVR was also evaluated in analyses.

### Software

#### Data collection

Flow cytometry data were collected on a Becton Dickinson X-20 Fortessa instrument using FacsDiva software.

### Data analyses

Initial flow cytometry analyses were performed using Flow.Jo.10.7.1 software. Singlets that were viable and CD45^+^ or CD45^+^CD3^+^CD4^+^ or CD45^+^CD3^+^CD8^+^ were exported as fcs files. Statistical analyses were conducted using R Version 4.0.5 and R Studio 2022.0.7.1. Several R packages were used for analyses : Tidyverse, Splitstackshape, flowCore, flowTrans, UMAP, and RPhenograph. The script files to perform all analyses and generate plots used in figures are available on Github.

### Data

#### Reporting on sex and gender

Sex was recorded at the beginning of the study based on self-reporting. Sex was not considered in the current study design. Sex is reported in Supplemental Fig. I.

### Population characteristics

Population characteristics are reported in Supplemental Fig. I.

### Recruitment

Participants were recruited from the community surrounding Wake Forest School of Medicine through media advertisement and community events sponsored by the Alzheimer’s Disease Research Center.

### Ethics oversight

All activities conducted in the Alzheimer’s Disease Research Center are approved and monitored by the Wake Forest School of Medicine Institutional Review Board (IRB#00,025,540). All experiments were performed in accordance with relevant guidelines and regulations.

### Life sciences study design

#### Sample size

Sample size was based on the availability of subjects for whom peripheral blood and cerebrospinal fluid could be collected.

### Data exclusions

No data were excluded.

### Replication

#### Randomization

No randomization was used.

## Blinding

Not applicable.

### Reporting for specific materials, systems and methods

#### Antibodies used

The antibodies used with our panels are listed below with dilutions used. All samples were stained with Fixable Viability Stain 450 (BD, 1:1000 dilution) prior to antibody staining. For intracellular cytokine staining samples, were stimulated with 50 ng/ml PMA (Sigma) and 5 ng/ml ION (Sigma) or 1 μg/ml peptide pool for 5 h in 1:1000 dilution GolgiPlug and GolgiStop at 37 °C in the tissue culture incubator. Samples were stained using the ebiosciences Fox3P intracellular staining kit.

**Table:** 

Target	Fluorophore	Clone	Vendor	Catalog number	Dilution
Pan immune panel (Fig. 1)					
CD45	BUV 395	HI30	BD	563,792	1:100
CD4	BUV 740	SK3	BD	612,448	1:50
Fixable viability stain	BV450	N/A	BD	562,247	1:1000
CD14	BV510	M582	BD	561,391	1:200
CD19	BV605	H1B19	Biolegend	302,244	1:100
CD123	BV650	7G3	BD	563,405	1:50
CD38	BV711	HIT2	BD	563,965	1:100
CD16	BV 786	3G8	BD	563,690	1:100
CD45RA	Fitc	5H9	BD	556,626	1:40
CD3	PerCP-Cy5.5	UCHT1	BD	560,835	1:50
CD61	PE	VI-PL2	BD	561,912	1:60
CD27	PE-Cy5	323	Invitrogen	15–0279-42	1:50
CD11c	Pe-Cy7	B-ly6	BD	561,356	1:200
Ki-67	AF 647	B56	BD	561,126	1:40
HLA-DR	AF 700	G46-6	BD	560,743	1:50
CD8	APC-Cy7	SK-1	BD	560,179	1:100
T cell memory phenotype panel (Fig. 2)					
CD45	BUV 395	HI30	BD	563,792	1:100
CD4	BUV 740	SK3	BD	612,448	1:50
Fixable viability stain	BV450	N/A	BD	562,247	1:1000
CD28	BV510	CD28.2	BD	563,075	1:25
CD62L	BV605	DREG-56	BD	562,720	1:40
CD69	BV650	FN-50	BD	563,835	1:40
CD38	BV711	HIT2	BD	563,965	1:200
CCR7	BV786	3D12	BD	563,710	1:40
CD45RA	Fitc	5H9	BD	556,626	1:10
CD3	PerCP-Cy5.5	UCHT1	BD	560,835	1:40
CD95	PE	DX2	BD	556,641	1:10
CD45RO	PE-CF594	UCHL1	BD	562,299	1:40
CD27	PECy5	0323	Invitrogen	15–0279-42	1:40
CD122	PECy7	TU27	Biolegend	339,014	1:20
HLA-DR	AF 700	G46-6	BD	560,743	1:40
CD8	APC-Cy7	SK-1	BD	560,179	1:100
T cell function and exhaustion panel (Figs. 3 and 4)					
CD45	BUV 395	HI30	BD	563,792	1:100
CD4	BUV 740	SK3	BD	612,448	1:50
Fixable viability stain	BV450	N/A	BD	562,247	1:1000
IFNγ	BV510	B27	BD	563,287	1:20
IL-2	BV605	MQ1-17H12	BD	564,165	1:40
T-bet	BV650	O4-46	BD	564,142	1:20
Ki-67	BV711	B56	BD	563,755	1:20
PD-1	BV786	RMP1-30	BD	748,264	1:20
CD45RA	Fitc	5H9	BD	556,626	1:10
CD3	PerCP-Cy5.5	UCHT1	BD	560,835	1:40
TCF-1	PE	S33-966	BD	564,217	1:60
LAG-3	PE-CF594	T47-530	BD	565,718	1:20
TNFα	PECy7	MAb11	BD	557,647	1:40
Tox	APC	REA473	Miltenyi	130-118-335	1:50
Granzyme B	AF700	GB11	BD	560,213	1:100
CD8	APC-Cy7	SK-1	BD	560,179	1:100

### Validation

All antibodies were obtained from commercial vendors and tested in titration experiments for optimal concentrations.

## Flow cytometry

### Methodology

#### Sample preparation

Cyropreserved PBMCs and CSF cells were used in this study. Cells were rapidly thawed the night before usage in 10% RPMI supplemented with benzonase, washed and then resuspended in media without benzonase. Cells were allowed to recover overnight before use. Cells were stained with the indicated antibodies above in FACS buffer (PBS + 2% FCS).

### Instrument

All samples were acquired on a BD Fortessa X-20 instrument using Diva software.

### Software

Initial flow cytometry analyses were performed in FloJo 10.7.1.

### Population abundance

#### Gating strategy

Cells were gated on viable singlets that were CD45^+^ and then the target of interest. Supplemental Figs. 3, 4 demonstrate the gating strategies used for Figs. 1, 2.

### Supplementary Information

Supplementary Figures.

## Data Availability

Flow cytometry data are available on Zenodo.com (https://zenodo.org/record/7570166). All code used to analyze data and create figures is available on Github.com(https://github.com/jaymgrayson/T_Cell_Exh_AD).
