# Supplementary material for: T cell exhaustion is associated with cognitive status and amyloid accumulation in Alzheimer’s disease
Source: Sci Rep. 2023 Sep 22;13:15779. doi: 10.1038/s41598-023-42708-8 (PMC10516910; doi:10.1038/s41598-023-42708-8)
Supplement: Supplementary file 1 — Supplementary Figures. [file 41598_2023_42708_MOESM1_ESM.pdf]

	ANCN (N=19)	APCN (N=15)	APMCI (N=9)	Overall (N=43)
**Sex**				
F	12 (63.2%)	14 (93.3%)	3 (33.3%)	29 (67.4%)
M	7 (36.8%)	1 (6.7%)	6 (66.7%)	14 (32.6%)
**Age (Years)**				
Mean (SD)	70.4 (7.87)	68.2 (5.90)	74.1 (7.23)	70.4 (7.26)
Median [Min, Max]	70.4 [57.0, 83.0]	66.9 [58.1, 79.0]	72.0 [63.4, 83.9]	68.5 [57.0, 83.9]
**BMI (lbs/in2)**				
Mean (SD)	28.0 (4.06)	27.4 (4.34)	24.3 (3.13)	27.0 (4.16)
Median [Min, Max]	26.8 [22.6, 37.4]	26.5 [19.8, 35.9]	25.2 [18.1, 27.4]	26.5 [18.1, 37.4]

Supplemental Figure I. Demographic characteristics of cohort used in this study.

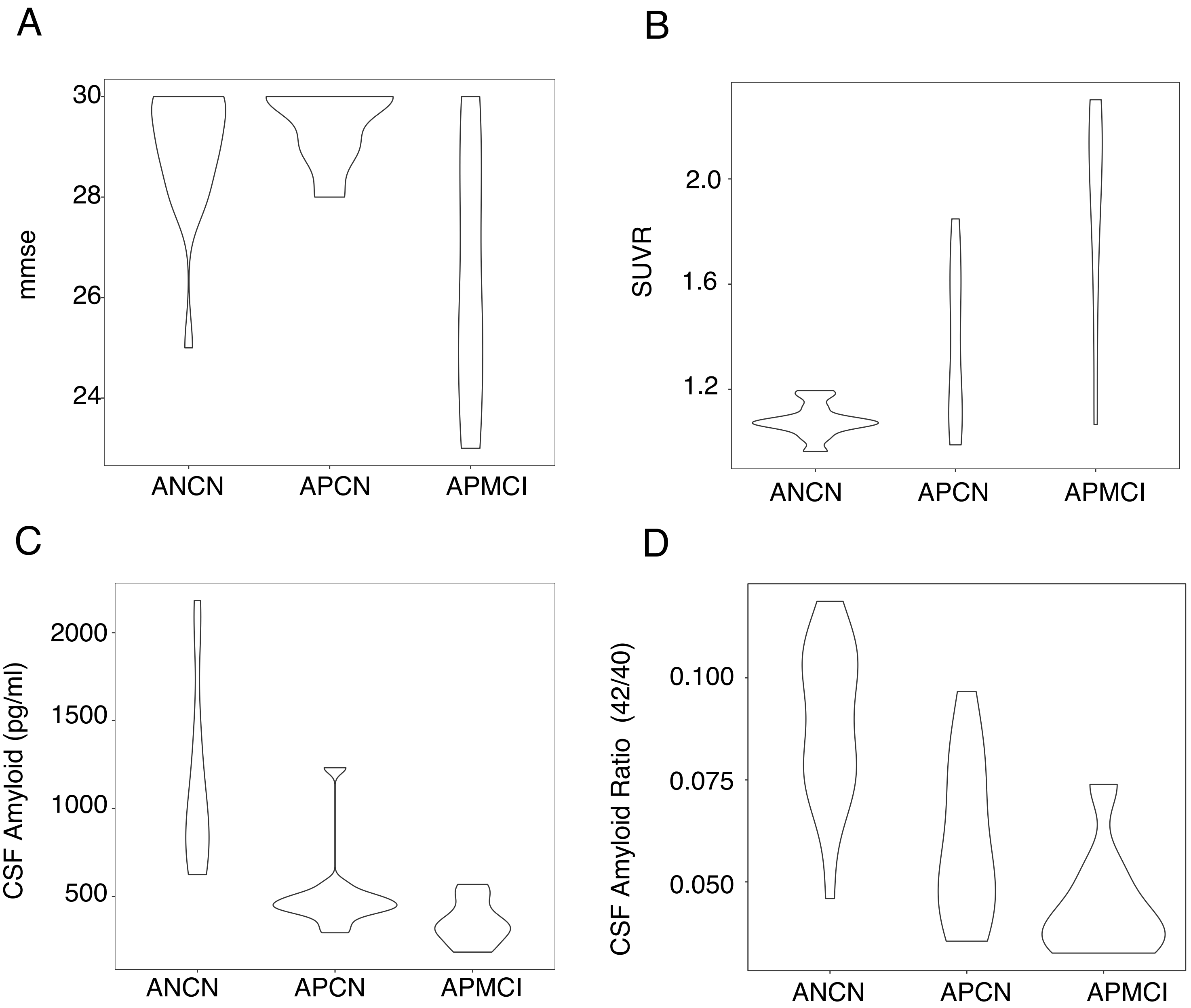

Supplemental Figure II. Cognitive and amyloid characteristics of cohort used in this study.

FS ROI #	Name
1002	L Caudate/Anterior Cingulate
1003	L caudal middle Frontal
1008	L Parietal Inferior
1010	L Isthmus Cingulate
1012	L Frontal Orbital
1014	L Frontal Orbital
1015	L Middle Temporal
1018	L Frontal Pars Opercularis
1019	L Frontal Pars Opercularis
1020	L Frontal Pars Opercularis
1023	L Post Cingulate
1025	L Precuneus
1026	L Rostral/Anterior Cingulate
1027	L rostral middle Frontal
1028	L Frontal Superior
1029	L Parietal Superior
1030	L Superior Temporal
1031	L Parietal Supramarginal gyrus
1032	L Frontal Orbital
2002	R Caudate/Anterior Cingulate
2003	R caudal middle Frontal
2008	R Parietal Inferior
2010	R Isthmus Cingulate
2012	Right Frontal Orbital
2014	Right Frontal Orbital
2015	R Middle Temporal
2018	R Frontal Pars Opercularis
2019	R Frontal Pars Opercularis
2020	R Frontal Pars Opercularis
2023	R Post Cingulate
2025	R Precuneus
2026	R Rostral/Anterior Cingulate
2027	R rostral middle Frontal
2028	R Frontal Superior
2029	R Parietal Superior
2030	R Superior Temporal
2031	R Parietal Supramarginal gyrus
2032	Right Frontal Orbital

Supplemental Figure III. Brain Regions of Interest Quantitated In Study

Immune Cell Subtype	Parameters
B Cells	$CD45^{+}CD123^{-}CD19^{+}$
Plasma Cells	$CD38^{+}HLA^{-}DR^{-}$
Naïve	$CD38^{-}HLA^{-}DR^{+}CD27^{-}$
Memory	$CD38^{-}HLA^{-}DR^{+}CD27^{+}$
T Cells	
$CD4^{+}$ T Cells	$CD45^{+}CD123^{-}CD19^{-}CD3^{+}CD4^{+}CD8\alpha^{-}$
Activated	$CD38^{+}$HLA-DR^{+}
Effector	$CD38^{-}$HLA-$DR^{-}CD27^{-}CD45RA^{+}$
Naïve	$CD38^{-}$HLA-$DR^{-}CD27^{+}$ $CD45RA^{+}$
Memory	$CD38^{-}$HLA-$DR^{-}CD27^{+}$ $CD45RA^{-}$
$CD8^{+}$ T Cells	$CD45^{+}CD123^{-}CD19^{-}CD3^{+}CD4^{-}CD8\alpha^{+}$
Activated	$CD38^{+}$HLA-DR^{+}
Effector	$CD38^{-}$HLA-$DR^{-}CD27^{-}CD45RA^{+}$
Naïve	$CD38^{-}$HLA-$DR^{-}CD27^{+}$ $CD45RA^{+}$
Memory	$CD38^{-}$HLA-$DR^{-}CD27^{+}$ $CD45RA^{-}$
Natural Killer (NK) Cells	$CD45^{+}CD123^{-}CD19^{-}CD3^{-}CD38^{+}CD16^{+}$
Monocytes	
Classical	$CD45^{+}CD123^{-}CD3^{-}CD19^{-}CD14^{+}CD11c^{+}$HLA-$DR^{+}$
Non-Classical	$CD45^{+}CD123^{-}CD3^{-}CD19^{-}CD14^{-}CD11c^{+}CD38^{-}$
Dendritic Cells	
Myeloid	$CD45^{+}CD123^{-}CD3^{-}CD19^{-}CD14^{-}CD11c^{+}CD38^{+}$HLA-$DR^{+}$
Plasmacytoid	$CD45^{+}CD123^{+}CD3^{-}CD19^{-}CD14^{-}CD11c^{-}CD38^{+}$HLA-$DR^{+}$

Supplemental Figure IV. Cellular gating strategies. Singlet, viable (FVS-) negative cells were gated as described above into the designated populations.

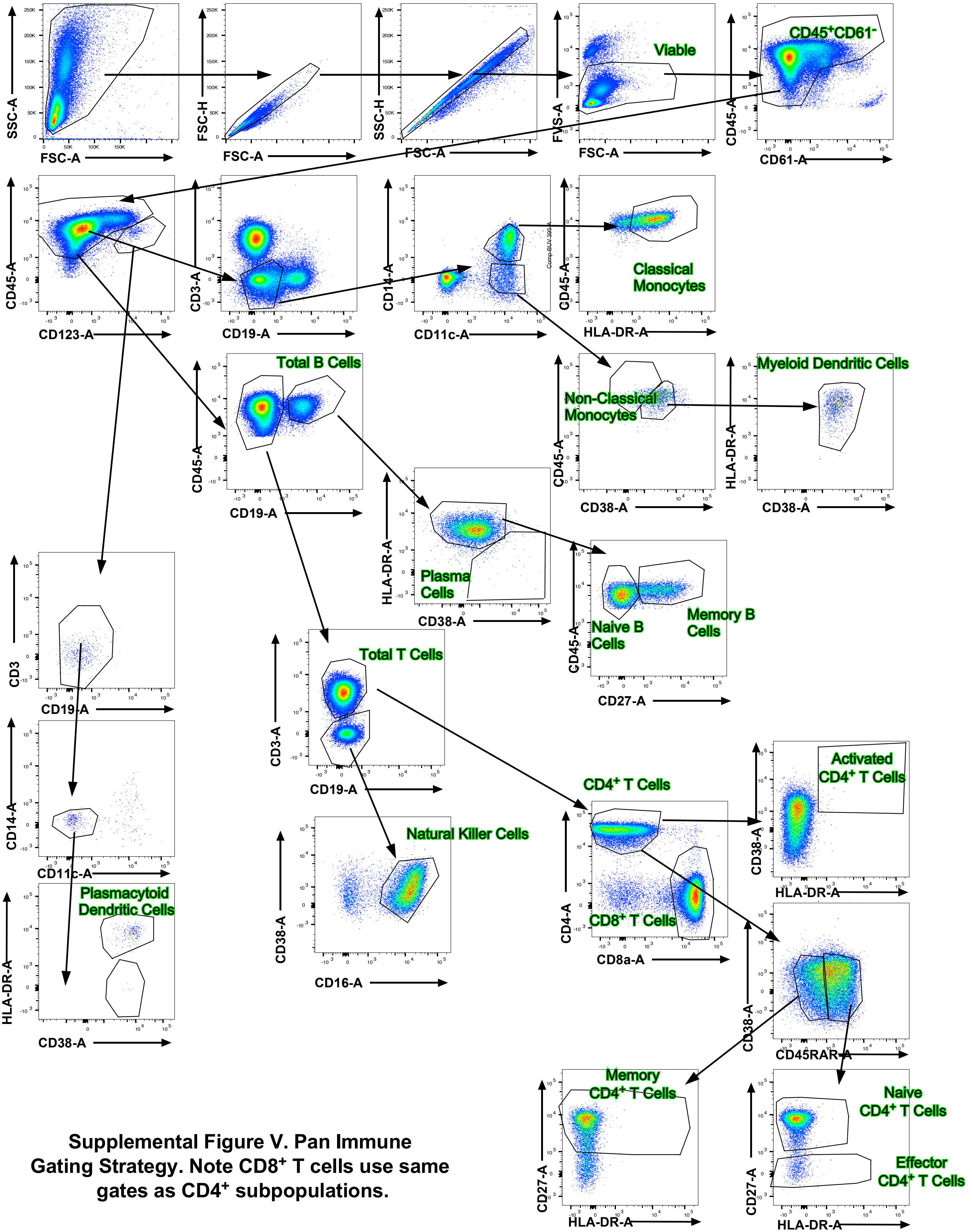

Supplemental Figure V. Pan Immune Gating Strategy. Note $CD8^+$ T cells use same gates as $CD4^+$ subpopulations.

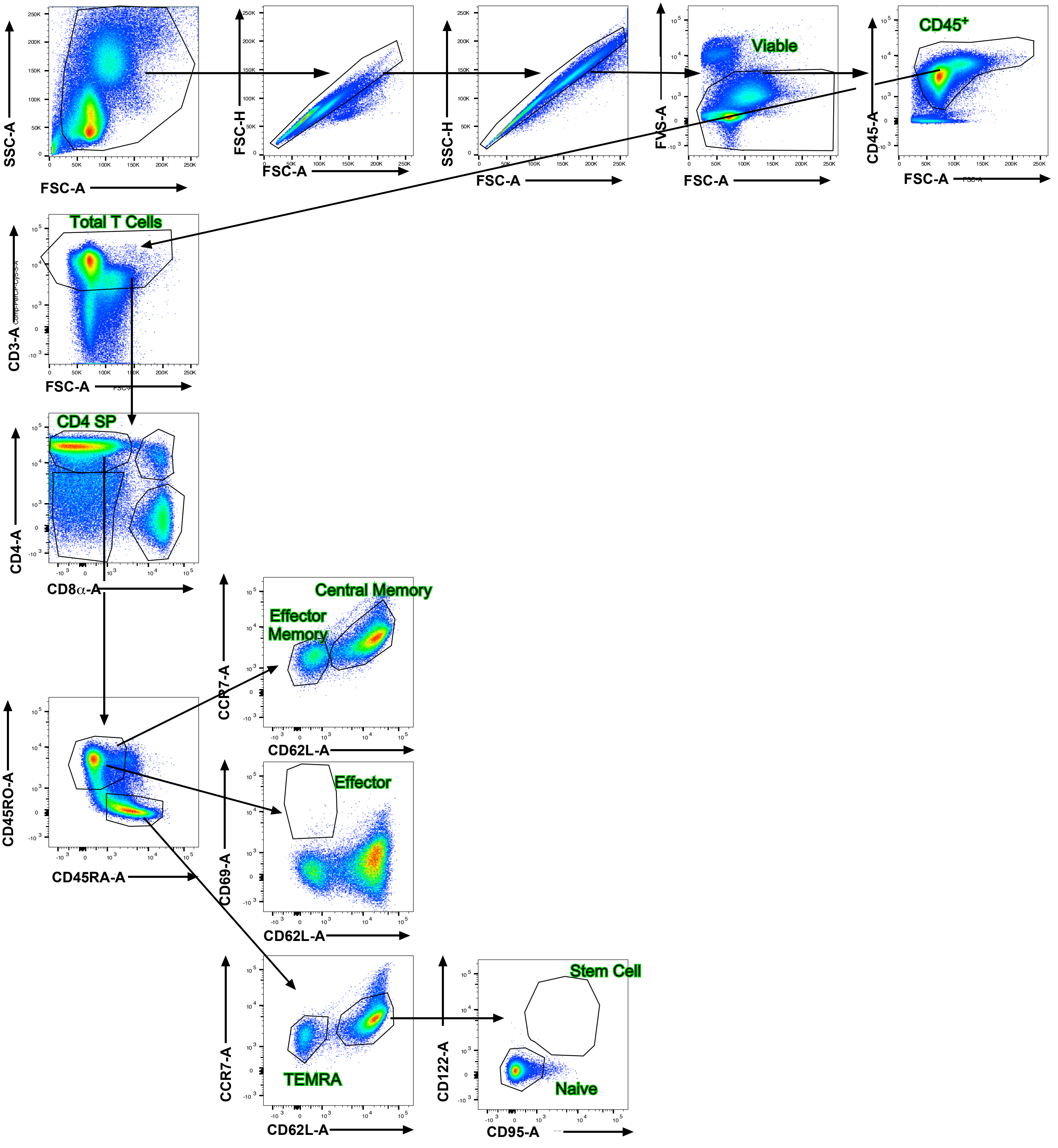

Supplemental Figure VI. T Cell Phenotype Gating Strategy. Note $CD8^{+}$ T cells use same gates as $CD4^{+}$ subpopulations.

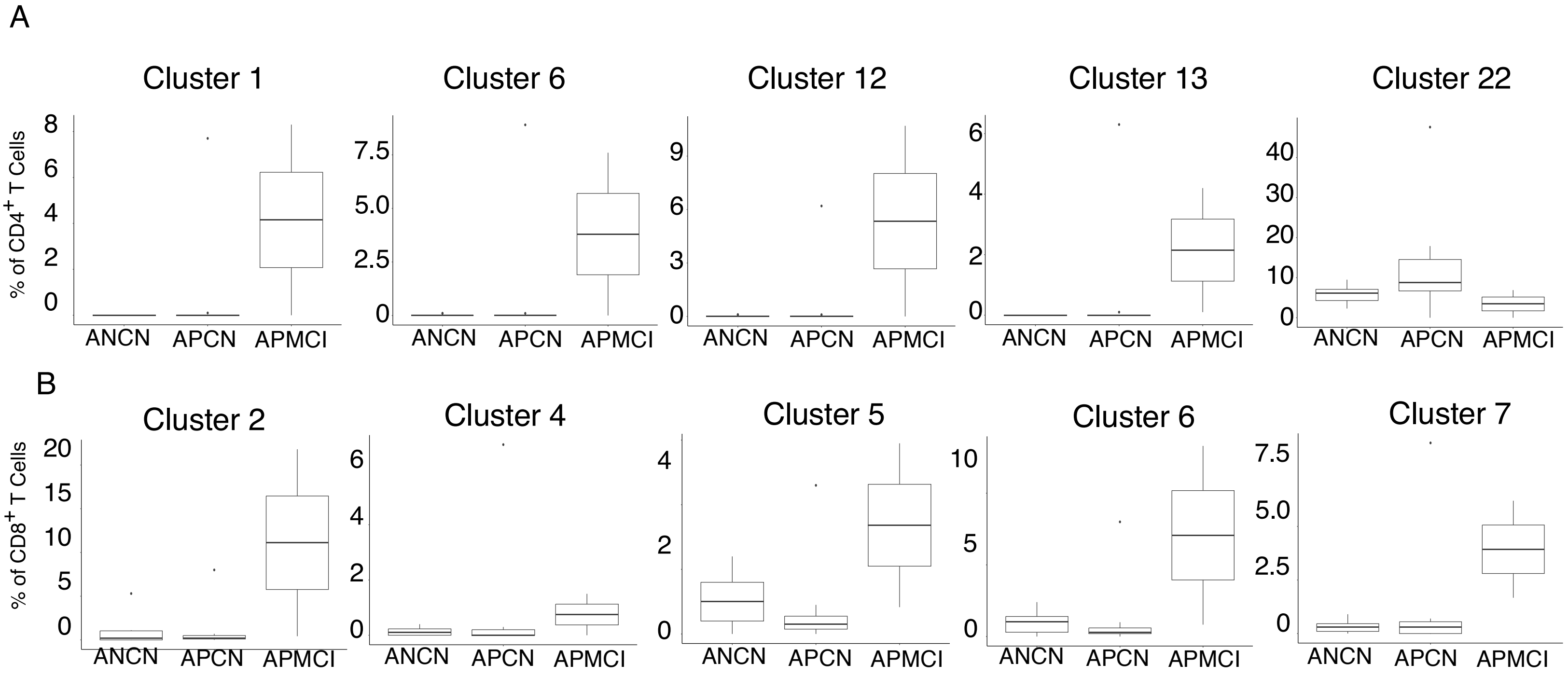

Supplemental Figure VII. CD4 and CD8 Functional Data For Female Participants.
